# PPARγ inhibition regulates the cell cycle, proliferation and motility of bladder cancer cells

**DOI:** 10.1111/jcmm.14280

**Published:** 2019-03-25

**Authors:** Songtao Cheng, Kaiyu Qian, Yejinpeng Wang, Gang Wang, Xuefeng Liu, Yu Xiao, Xinghuan Wang

**Affiliations:** ^1^ Department of Urology Zhongnan Hospital of Wuhan University Wuhan China; ^2^ Department of Biological Repositories Zhongnan Hospital of Wuhan University Wuhan China; ^3^ Human Genetics Resource Preservation Center of Hubei Province Wuhan China; ^4^ Laboratory of Precision Medicine Zhongnan Hospital of Wuhan University Wuhan China; ^5^ Department of Pathology Lombardi Comprehensive Cancer Center, Georgetown University Medical School Washington District of Columbia; ^6^ Medical Research Institute, Wuhan University Wuhan China

**Keywords:** bladder cancer, cell cycle, GW9662, motility, PPARγ

## Abstract

Peroxisome proliferator‐activated receptor gamma (PPARγ) is a member of the nuclear receptor family of ligand‐activated transcription factors and plays an important role in regulating cell proliferation, inflammation and lipid and glucose homeostasis. Our results revealed that PPARγ was up‐regulated in human bladder cancer (BCa) tissues both at transcriptional and translational levels. Moreover, down‐regulation of *PPARγ *mRNA or inhibition of PPARγ function (using GW9662, antagonist of PPARγ) could significantly suppress the proliferation of BCa cells. Furthermore, the cell cycle arrested in G0/G1 phase was also induced by the down‐regulated PPARγ possibly through AKT‐mediated up‐regulation of p21/p27, whereas no significant transformation of apoptosis was observed. In addition, knockdown or inhibition of PPARγ might reduce the invasion and migration of BCa cells by affecting epithelial‐mesenchymal transition‐related proteins through AKT/GSK3β signalling pathway. Additionally, in vivo studies showed that BCa cell proliferation was significantly suppressed by GW9662. In conclusion, our results indicated that PPARγ might be crucial for BCa tumorigenesis by interfering with the motility and viability of BCa cells.

## 1 INTRODUCTION

Bladder cancer (BCa), with a gradually increasing global incidence in the past 30 years, is now the ninth most common cause of cancer, occurs more frequently among males than females (sex ratio worldwide of 3.5:1) and is relatively common in more developed areas, representing 60% of all cases.^1^ Bladder cancer is also one of the most common malignancies in the urinary system, and 70% of newly diagnosed patients are non‐muscle‐invasive BCa (NMIBC), indicating that cancer cells are confined to the lamina propria and urothelial layers of the bladder.^2^ Currently, the preferred therapeutic method for NMIBC is transurethral resection of the bladder tumour, followed by intravesical instillations of chemotherapy or immunotherapy.^3^ According to an analysis of 2596 patients with NMIBC, the 5‐year recurrence rate reached 31% to 78% and the risk of progression to muscle‐invasive BCa (MIBC) ranged from 1% to 45%.^4^ The gold standard therapeutic method for MIBC is radical cystectomy, which is still associated with many unfavourable outcomes.^5^, ^6^ Furthermore, even after receiving radical cystectomy, the BCa recurrence and distant metastasis rate remain at approximately to 50%, and the 5‐year survival rate is only 50%‐66%.^7^, ^8^ Therefore, more effective therapeutic strategies are needed for BCa treatment.

Peroxisome proliferator‐activated receptor gamma (PPARγ), a member of the nuclear receptor family of ligand‐activated transcription factors that regulates gene expression via the formation of protein heterodimers with the retinoic X receptor (RXR), is a key nuclear receptor regulating adipocyte differentiation and glucose homeostasis.^10^ Peroxisome proliferator‐activated receptor gamma has been reported to interact with multiple signalling pathways, including BCL2, NFκB, p53, p21, STAT, cyclooxygenase‐2 (COX‐2) and Cyclin D1.^11^ Additionally, PPARγ is highly expressed in certain cancer cell. However, its function in tumour progression remains controversial.^10^, ^12^ Some previous studies have suggested that the activation of PPARγ can prevent tumours in tissues such as the lung, colon, prostate, breast and others,^13^, ^14^ while others have revealed that activated PPARγ is oncogenic.^18^, ^19^ Therefore, the role of PPARγ in tumorigenesis needs to be further clarified. In this study, we aimed to identify the effects of PPARγ in BCa.

Ligands of PPARγ contain endogenetic fatty acids and thiazolidinedione (TZD) classes of anti‐diabetic drugs such as pioglitazone, which has weak PPARα activity, and rosiglitazone, which is highly selective for PPARγ.^22^ Some clinical trials have shown that PPARγ agonists are associated with increased risks of bone fractures^23^, ^24^ heart failure^25^ and BCa.^26^ For instance, an increased incidence of BCa has been observed, as a side effect in rodent toxicity studies using PPAR agonists to examine anti‐diabetic effects,^18^ whereas other studies indicated no increased risk.^27^, ^28^ Recently, a comprehensive retrospective study showed an increased hazard ratio with long‐term, high‐dose treatment with pioglitazone in BCa.^29^ In addition, some compounds that antagonize PPARγ have been reported to inhibit the proliferation or invasiveness of human tumour cell lines, such as colon carcinoma, renal cell carcinoma, oesophageal carcinoma, hepatocellular carcinoma and others^20^, ^21^, ^30^ Therefore, in our study, we chose GW9662, the selective and irreversible PPARγ antagonist,^31^ to treat the BCa cells and detected the alterations in viability and motility.

Our group has focused on novel biomarkers and pathways for the long‐term prevention of BCa progression. Our previous studies have suggested that HJURP may regulate apoptosis and proliferation in BCa cells through the PPARγ‐SIRT1 feedback loop^32^ and that simvastatin can induce cell cycle arrest in G1/G0 phase and inhibit proliferation in BCa cells via the PPARγ signalling pathway.^33^ In this study, we aimed to investigate the function of *PPARγ* gene and its effects on BCa tumorigenesis.

## 2 MATERIALS AND METHODS

### 2.1 Ethical statement for human bladder tissues

As described by Wang et  al^33^ and Cao et  al^34^ from our group, paracancerous and BCa tissues (n = 9) were obtained from patients after surgery at Zhongnan Hospital of Wuhan University, and normal bladder tissues were obtained from donors who experienced accidental death. The tissue samples were fixed in 4% paraformaldehyde (PFA) for subsequent immunofluorescence (IF) staining or snap‐frozen and stored in liquid nitrogen for subsequent RNA isolation. Informed consent was obtained from all subjects, and the study was conducted in accordance with the Declaration of Helsinki. The use of human bladder tissues for IF staining analysis and RNA isolation was approved by the Ethics Committee at Zhongnan Hospital of Wuhan University (approval no. 2015029).

### 2.2 BCa cell lines

The human BCa cell lines 5637 (Cat. #TCHu 1), T24 (transitional cell carcinoma, Cat. #SCSP‐536) and UM‐UC‐3 (Cat. #TCHu217) were acquired from the Chinese Academy of Sciences in Shanghai, China. The cell lines were authenticated by the China Center for Type Culture Collection in Wuhan, China. The 5637 and T24 cells were maintained in RPMI‐1640 medium (Gibco, Shanghai, China) and UM‐UC‐3 cells were maintained in DMEM (Gibco) supplemented with 1% penicillin G sodium/streptomycin sulphate and 10% foetal bovine serum (FBS) (Gibco, Melbourne, Australia) in a humidified atmosphere composed of 5% CO_2_ and 95% air at 37ºC.

### 2.3 RNA expression analyses

#### 2.3.1 Total RNA isolation from bladder cells and tissues

Total RNA was extracted from BCa cells and bladder tissues using the Qiagen RNeasy Mini Kit (Cat. #74101; Qiagen, Hilden, Germany) and QIAshredder from Qiagen (Cat. #79654, Qiagen) according to the manufacturer's instructions. Quantity control of the isolated RNA was assessed using a NanoDrop^®^ ND‐2000 UV‐Vis spectrophotometer (Thermo Scientific, Madison, WI, USA).

#### 2.3.2 Reverse transcription and quantitative real‐time PCR

The cDNA was synthesized from 1 μg of total RNA using the RevertAid Ace quantitative real‐time PCR (qPCR RT) kit (Toyobo, Shanghai, China). For qRT‐PCR analysis, 1 μg of cDNA was used in each reaction in a final volume of 20 μL with iQ™ SYBR^®^‑Green Supermix (Bio‐Rad, Shanghai, China). All the primer sequences with annealing temperatures are listed in Table 1. The cycle number threshold (CT) values were normalized to glyceraldehyde 3‐phosphate dehydrogenase (GAPDH), and calculated as follows 35: relative gene expression = 2^−ΔΔct^, Δct = ct_target gene_ − ct*_GAPDH_*, for BCa cells ΔΔct = Δct_siRNA‐treated_ − Δct_siRNA‐control_, for bladder tissues ΔΔct = Δct_BCa tissues_ − Δct_paracancerous tissues_.

**Table 1:** List of primers for quantitative real‐time PCR

Gene	Symbol	Forward primer (5′‐3′)	Reverse primer (5′‐3′)	Annealing temperature (°C)	Length (bp)
Peroxisome proliferator‐activated receptor gamma	PPARγ	ATGACAGACCTCAGACAGATTG	AATGTTGGCAGTGGCTCAG	60	148
Glyceraldehyde‐3‐phosphate dehydrogenase	GAPDH	TGCACCACCAACTGCTTAG	GATGCAGGGATGATGTTC	60	176

### 2.4 Cell culture experiments

#### 2.4.1 Knockdown of *PPARγ* in BCa cells

Negative control small interfering RNA (siRNA) and *PPARγ* target siRNA were synthesized by Genepharma (Shanghai, China). The sense sequence of *PPARγ* target siRNA (*si‐PPARγ*) was as follows: *Si‐1*, 5'‐ GGUUGCAGAUUACAAGUAUTT‐3ʹ; *Si‐2*, 5ʹ‐GCGGAGAUCUCCAGUGAUATT‐3ʹ; *Si‐3*, 5ʹ‐GCUGGCCUCCUUGAUGAAUTT‐3ʹ; *Si‐4*, 5ʹ‐AGAACAAUCAGAUUGAAGCTT‐3ʹ. The sense sequence of *si‐control* was 5ʹ‐UUCUCCGAACGUGUCACGUTT‐3ʹ. Cells were transfected with *si‐PPARγ* and *si‐control* using lipoJetTM (SignaGen, China) according to the manufacturer's protocol when the cells had grown to 60%. After transfection for 48 hours, PPARγ alterations were detected by Western blot and qRT‐PCR analyses.

#### 2.4.2 PPARγ antagonist treatment

Bladder cancer cells were first incubated for 24 hours and subsequently treated with the PPARγ antagonist GW9662 (Sigma‐Aldrich, Cat. #M6191) at 0, 0.1, 10, 20 and 40 μmol/L for 24, 48, 72 and 96 hours. After selecting the appropriate concentrations, all the following relevant experiments were conducted with the cells pre‐treated with GW9662 at 0, 10 and 20 μmol/L for 48 hours. GW9662 was initially dissolved in dimethyl sulfoxide (DMSO) as a stock solution at a concentration of 50 mmol/L, and DMSO was added to the 0 group at a concentration of 0.1% as a control.

#### 2.4.3 Clonogenic survival assay

To six‐well plates were added 800 UM‐UC‐3 cells/well, 1000 T24 cells/well and 3000 5637 cells/well for growth into colonies for 7‐10 days. After removing the medium, fixing the cells with 4% PFA, and staining with crystal violet for 30 minutes, imaging and counting were performed.

#### 2.4.4 Methyl thiazolyl tetrazolium assay

Into 96‐well plates were pipetted 3000 BCa cells in 200 μL medium for growth for 48 hours. To each well was added in 20 μL methyl thiazolyl tetrazolium (MTT) and incubated for 4 hours at 37°C, discarding the medium and dissolving the formazan precipitate in 150 μL DMSO. The absorbance at 490 nm was then detected using a microplate reader (Cat. no. SpectraMax M2; Molecular Devices, Berkeley, CA, USA).

#### 2.4.5 Transwell chamber invasion and migration assay

To the upper transwell chamber (Corning, New York, NY, USA) was seeded 4‐8 × 10^4^ BCa cells in 200 μL serum‐free medium, and 600 μL medium containing 10% FBS was added to the lower chamber to induce cell migration. After incubation for 24 hours at 37°C and removal of the cells in the upper chamber using cotton swabs, the migrated cells on the lower side of the chamber were fixed with 4% PFA, stained with 0.1% crystal violet and imaged and counted cells under a phase contrast microscope. For the invasion assay, the transwell chambers were percolated with ECM Matrix gel solution (Sigma‐Aldrich) for 1 hour at 37°C, and 1 × 10^5^ cells were seeded to incubate for 24‐48 hours as described previously. Subsequent procedures were equivalent to the migration assays.

#### 2.4.6 Flow cytometry analysis of alterations of apoptosis and the cell cycle

After collection and washing with cold PBS, BCa cells were resuspended in 1× DNA Staining Solution containing permeabilization solution and propidium iodide (Cat. #CCS012, Multi Sciences, Hangzhou, China) and then incubated in the dark for 30 minutes at 37°C. Flow cytometry (Cat. #CytoFLEX; Beckman, Carlsbad, CA, USA) was used to analyse the cell cycle. According to the manufacturer's instruction, cell apoptosis analysis was conducted using the annexin V‐fluorescence isothiocyanate (FITC)/PI apoptosis detection kit (Cat. #558547, BD Biosciences, San Jose, CA, USA) by flow cytometry.

### 2.5 Protein Analyses

#### 2.5.1 Isolation of total protein and Western blot analysis

Bladder cancer cells were lysed and sonicated on ice in radioimmunoprecipitation assay buffer including phosphatase inhibitor and protease inhibitor (Sigma‐Aldrich) for approximately 30 minutes. They were then centrifuged at 12 000 *g* for 15 minutes, and the supernatant was collected. The Bradford protein assay (Bio‐Rad, Munich, Germany) was used to determine the protein concentration using bovine serum albumin (BSA) as the standard. Protein was separated using 7.5%‐10% SDS‐PAGE gels and transferred to a polyvinylidene fluoride membrane (Millipore, Billerica, MA, USA), which was then blocked in 5% fat‐free milk (BD biosciences) at room temperature for 2 hours and incubated with primary antibodies overnight (Table 2) and secondary antibodies for 2 hours (Table 3). Bands were detected using an enhanced chemiluminescence (ECL) kit (Bio‐Rad, Hercules, CA, USA) and visualized with Biomax MR films (Kodak, Rochester, NY).

**Table 2:** List of primary antibodies

Antigens	Species antibodies raised in	Dilution (IF)	Dilution (WB)	Supplier
Glyceraldehyde 3‐phosphate dehydrogenase (GAPDH), human	Mouse, monoclonal	—	1:2000	Santa Cruz Biotechnology Inc, USA, cat. no. sc‐365062
E‐Cadherin, human	Rabbit, monoclonal	1:200	1:500	Cell Signaling Technology, USA, cat. no. 3195
N‐Cadherin, human	Rabbit, monoclonal	1:200	1:1000	Cell Signaling Technology, USA, cat. no. 13116
Ki‐67, human	Rabbit, monoclonal	1:100	—	Novus Biologicals, USA, cat. no. NBP2‐19012
Phospho‐AKT(Ser473), human	Rabbit, monoclonal	—	1:2000	Cell Signaling Technology, USA, cat. no. 4060
AKT (pan), human	Rabbit, monoclonal	—	1:2000	Cell Signaling Technology, USA, cat. no. 4691
Phospho‐GSK‐3β (Ser9), human	Rabbit, monoclonal	—	1:2000	Cell Signaling Technology, USA, cat. no. 5558
GSK3β, human	Rabbit, monoclonal	—	1:2000	Cell Signaling Technology, USA, cat. no. 12456
P21, human	Rabbit, monoclonal	—	1:1000	Abcam, UK, cat. no. ab109520
P27, human	Rabbit, monoclonal	—	1:1000	Abcam, UK, cat. no. ab32034
Slug, human	Rabbit, monoclonal	—	1:1000	Cell Signaling Technology, USA, cat. no. 9585
Cyclin D1, human	Rabbit, monoclonal	—	1:1000	Cell Signaling Technology, USA, cat. no. 2978
CDK2, human	Rabbit, monoclonal	—	1:1000	Cell Signaling Technology, USA, cat. no. 2546
CDK4, human	Rabbit, monoclonal	—	1:1000	Abcam, UK, cat. no. ab108357
CDK6, human	Rabbit, monoclonal	—	1:1000	Abcam, UK, cat. no. ab124821
MMP‐2, human	Rabbit, monoclonal	—	1:500	Cell Signaling Technology, USA, cat. no. 13132
MMP‐9, human	Rabbit, monoclonal	—	1:1000	Cell Signaling Technology, USA, cat. no. 13667
Vimentin, human	Rabbit, monoclonal	—	1:1000	Cell Signaling Technology, USA, cat. no. 5741
PPARγ, human	Rabbit, monoclonal	1:100	1:500	Abcam, UK, cat. no. ab45036

**Table 3:** List of secondary antibodies and counterstaining of nuclei

Secondary detection system used	Host	Method	Dilution	Supplier
Anti‐mouse IgG (H + L)‐HRP	Goat	WB	1:10 000	Sungene Biotech, China, cat. no. LK2003
Anti‐rabbit IgG (H + L)‐HRP	Goat	WB	1:10 000	Sungene Biotech, China, cat. no. LK2001
Anti‐rabbit IgG (H + L), F(ab’)2 Fragment (Alexa Fluor^®^ 488 Conjugate)	Goat	IF	1:100	Cell Signaling Technology, USA, cat. no. 4412
Anti‐rabbit IgG (H + L), F(ab’)2 Fragment (Alexa Fluor^®^ 555 Conjugate)	Goat	IF	1:100	Cell Signaling Technology, USA, cat. no. 4413
Hoechst 33342 nucleic acid staining (DAPI)	‐	IF	1:750	Molecular Probes/Invitrogen, Carlsbad, CA, USA, cat. no. A11007

#### 2.5.2 Immunofluorescence staining

Bladder cancer cells were seeded on the coverslips, washed with cold phosphate‐buffered saline (PBS, pH 7.4) twice, fixed with 4% PFA, treated with 0.1% Triton X‐100 solution and blocked in goat serum for 30 minutes. Then, the cells were treated with primary antibodies and FITC‐labelled or Cy3‐labelled secondary antibody. Nuclei were all stained with 4',6‐diamidino‐2‐phenylindole (DAPI) (2 μg/mL). The images were obtained using a fluorescence microscope (Olympus, Cat. #IX73) or confocal laser scanning microscope (Nikon Ltd., Tokyo, Japan). For human bladder and xenograft mouse tissues fixed with 4% PFA and embedded in paraffin (Paraplast; Sigma‐Aldrich) using a tissue processor (Cat. #STP 120, Thermo Fisher Scientific, Loughborough, UK), 5‐μm‐thick tissue slices were cut using a rotation microtome (Cat. #HM325, Thermo Fisher Scientific, Walldorf, Germany). The subsequent process was the same as described previously (antibodies listed in Tables 2 and 3).

#### 2.5.3 Haematoxylin and eosin staining

All the tissue paraffin sections from xenotransplanted cancer samples were stained with haematoxylin and eosin (H&E). They were then serially deparaffinized and rehydrated in xylene, 100% ethanol, 96% ethanol, 80% ethanol, 70% ethanol and H_2_O. The sections were subsequently stained with 10% haematoxylin (Sigma‐Aldrich) for 7 minutes, and 1% eosin (Sigma‐Aldrich) with 0.2% glacial acetic acid was used to stain the cytoplasm after washing to reveal the nuclei. The tissue sections were washed and dehydrated successively in 70%, 80%, 96% and 100% ethanol, followed by 10 minutes in xylene. Images of the sections were obtained using an inverted phase contrast microscope (Cat. #DMI 1; Leica, Wetzlar, Germany).

### 2.6 Xenograft mouse model

Male BALB/c nude mice aged 3 weeks were obtained from Beijing HFK Bioscience Co., Ltd. in Beijing, China. After a 1‐week adaptation period, the mice that were maintained in a temperature and humidity‐controlled and specific pathogen‐free environment in the laboratory animal facility of Zhongnan Hospital of Wuhan University, were injected into the right flank with 3 × 10^6^ T24 cells dispersed in 0.2 mL PBS. Ten days later, mice with transplanted tumours that were approximately 3 × 3 mm were randomly assigned to one of two groups (n = 5). GW9662 (1 mg/kg body weight) and vehicle were injected intraperitoneally every other day for 2 weeks. Tumour size was measured every 2 days using a caliper, and the values were calculated as follows: tumour volume (mm^3^) = length × width^2^ × 0.5 mm^3^. After the mice were killed, all the tumours tissues were dissected and fixed in 4% PFA preparing for subsequent H&E staining and IF staining.

### 2.7 Statistical analyses

All experiments were performed at least three times, and representative data were from three iterations. One‐way ANOVA and two‐tailed Student's *T* test were used to evaluate the statistical significance of differences between subgroups. All the statistical analyses were conducted with spss 16.0, and the cut‐off level was set at probability values of *P* < 0.05.

## 3 RESULTS

### 3.1 Up‐regulation of PPARγ in BCa tissues compared with normal and paracancerous tissues

**Figure 1:**
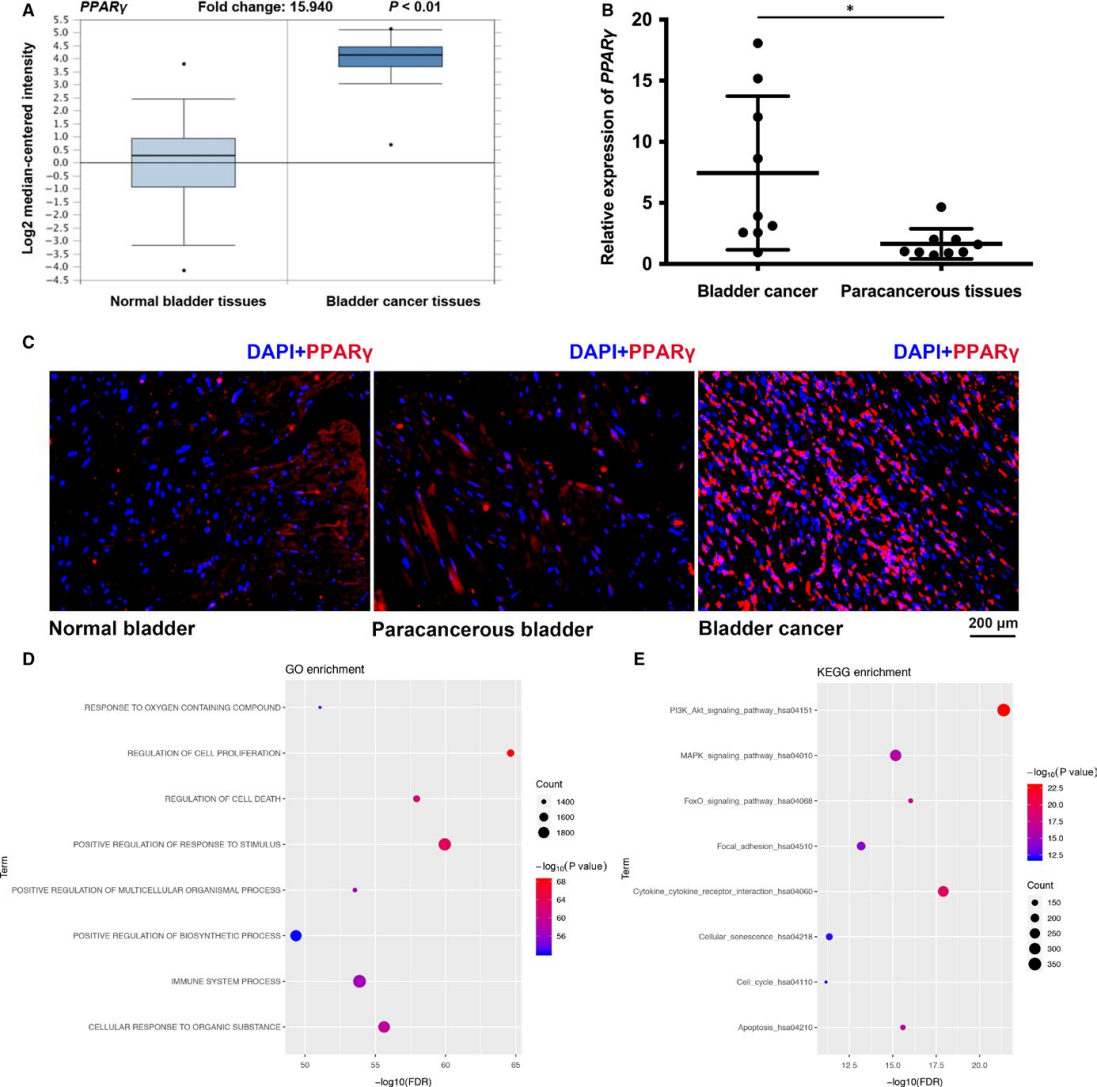
Up‐regulated PPARγ in BCa tissues. A, Up‐regulated mRNA expression of *PPARγ* in BCa analysed from the Oncomine database. The* P*‐value and fold change are indicated. B, The quantitative real‐time PCR results indicated that the gene expression of *PPARγ* was significantly up‐regulated in BCa tissues compared with the matched paracancerous tissues. The reference gene is *GAPDH*. *P < 0.05. C, Representative immunofluorescence staining of PPARγ (red) in normal bladder tissues, BCa tissues and paracancerous tissues. Nuclei were labelled with 4',6‐diamidino‐2‐phenylindole (DAPI; blue), and the scale bar is indicated. D, GO enrichment and (E) Kyoto Encyclopedia of Genes and Genomes pathway enrichment of *PPARγ *in miRNA Cancer MAP database

First, we searched in the Oncomine database, which showed that the expression of PPARγ was significantly up‐regulated in BCa tissues at the transcriptional level (Figure 1A). Our qRT‐PCR results also showed increasing expression of *PPARγ* in BCa in comparison to paracancerous BCa tissues (n = 9, Figure 1B). Furthermore, IF staining confirmed a strong up‐regulation of PPARγ protein in BCa tissues in comparison to normal bladder and paracancerous tissues (Figure 1C). To further detect the function of *PPARγ, *gene function enrichment was performed using miRNA Cancer MAP (http://cis.hku.hk/miRNACancerMAP/index.php).^36^ The Cancer Genome Atlas Bladder urothelial carcinoma datasets were chosen and the cut‐off *P*‐value was set at 0.01. Gene ontology (GO) enrichment analysis revealed that *PPARγ *was involved in regulation of cell proliferation, positive regulation of biosynthetic process, regulation of cell death and so on (Figure 1D; Table S1). Kyoto Encyclopedia of Genes and Genomes (KEGG) pathway analysis indicated that *PPARγ *was related to cell cycle, focal adhesion, apoptosis, PI3K‐AKT signalling pathway and so on (Figure 1E; Table S2).

### 3.2 Knockdown of PPARγ impaired the viability of BCa cells

**Figure 2:**
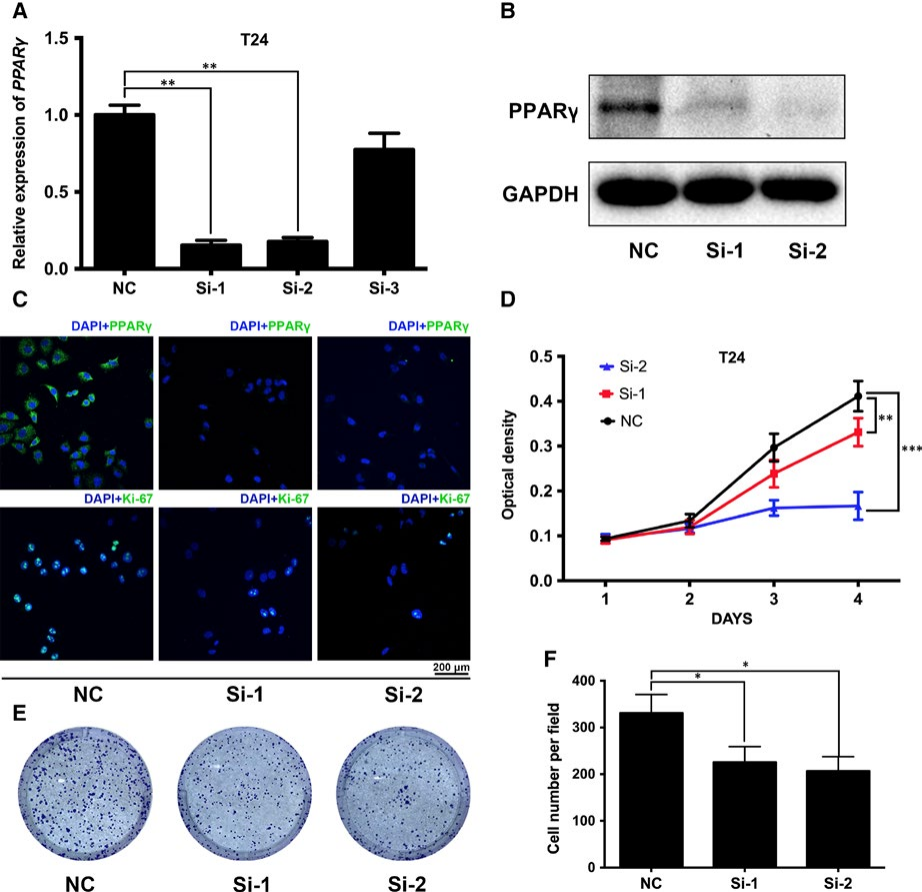
Down‐regulation of PPARγ inhibited the proliferation of BCa cells. A, Quantitative real‐time analysis validated the efficacy of knockdown by using distinct *PPARγ* target‐specific siRNA at the transcriptional level in BCa T24 cells. B, Western blot and (C) immunofluorescence (IF) staining revealed a reduced protein abundance of PPARγ by siRNA treatment compared with the NC group. D, The methyl thiazolyl tetrazolium assay was used to test the viability of BCa T24 cells transfected with NC (black line) or siRNA (red and blue lines), and the results were in accordance with the IF staining of Ki‐67. E, The influence of PPARγ down‐regulation on cell survival was measured by the clonogenic survival assay and (F) statistical analysis of three independent results. *P < 0.05, **P < 0.01, ***P < 0.001

To detect the effects of *PPARγ* on biological behaviours in BCa, we chose four distinct *PPARγ* target siRNAs to transfect BCa T24 cells to create a *PPARγ*‐deficient cell model. After transfection for 48 hours, qRT‐PCR (Figure 2A; Figure S1A), Western blot (Figure 2B; Figure S1B) and IF staining (Figure 2C; Figure S1F) analysis were used to validate the knockdown efficiency. The results showed a significant reduction of PPARγ at the mRNA and protein level in the *Si‐1‐*, *Si‐2‐* and *Si‐4‐*treated group compared with the NC (*control‐siRNA*) group. Then, T24 cells were treated with *control‐siRNA* and *PPARγ‐siRNA* for 48 hours, and an inhibition of proliferation of *PPARγ‐siRNA*‐treated T24 cells was observed in comparison to the NC group, as detected by the MTT assay (Figure 2D; Figure S1D). The clonogenic survival results showed a distinct reduction in colony‐forming efficiency (Figure 2E; Figure S1C), which was statistically significant (Figure 2F). Moreover, Ki‐67, an important marker of cell proliferation, was significantly reduced in the siRNA‐treated group compared with the NC group (Figure 2C).

### 3.3 Down‐regulation of PPARγ inhibited cell motility with alterations in AKT/GSK3β/EMT‐related proteins

**Figure 3:**
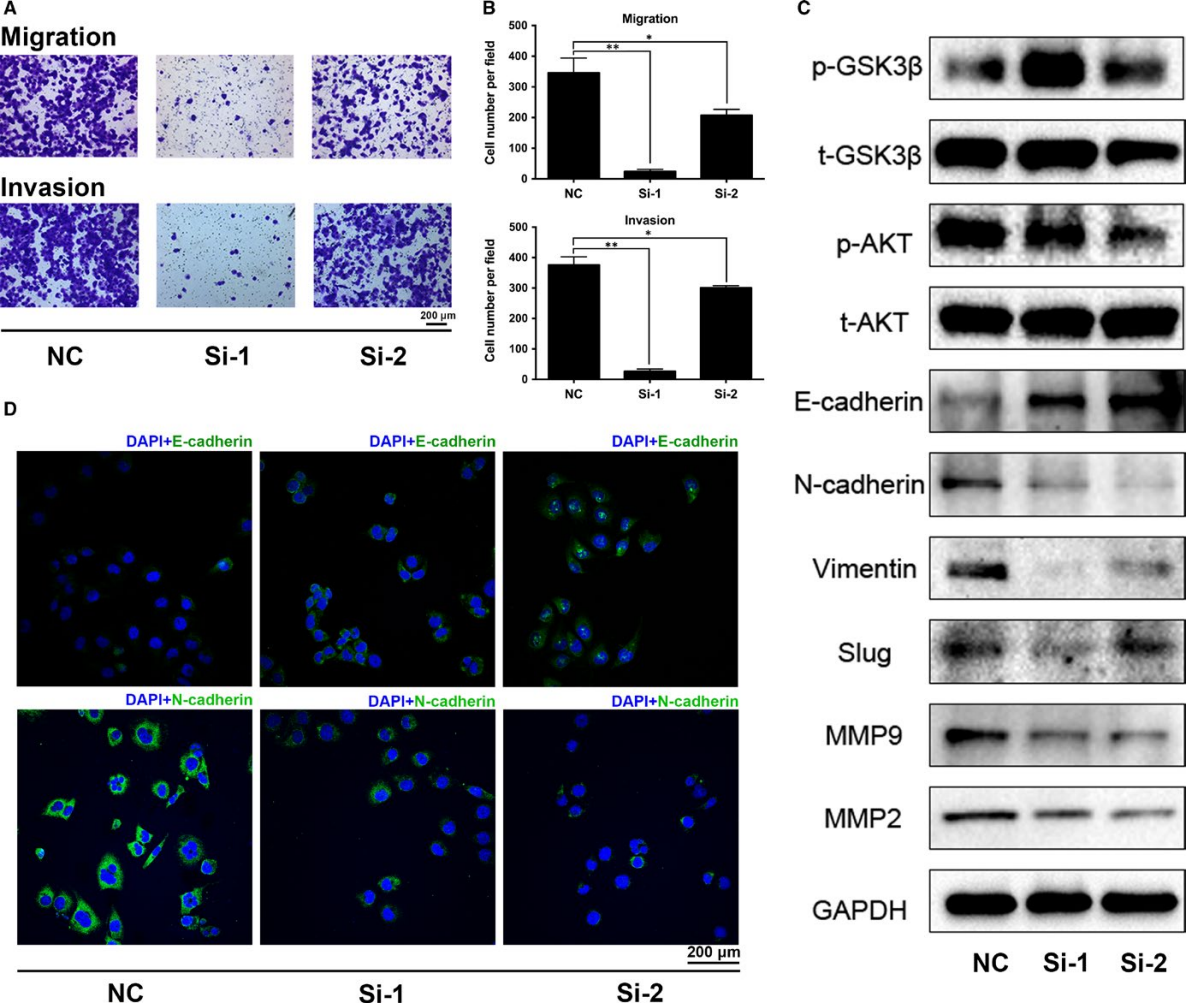
Knockdown of *PPARγ* altered epithelial‐mesenchymal transition (EMT) and AKT/GSK3β signalling pathway proteins in T24 cells. A, Transwell invasion and migration assay for T24 cells with or without siRNA. The scale bar is 200 μm. B, Statistical analysis of the transwell assay suggested significantly decreased migration and invasion of the cells after siRNA treatment. C, Western blot results for proteins related to EMT regulation revealed a strong reduction of N‐cadherin, vimentin, slug, MMP2 and MMP9, while E‐cadherin was up‐regulated after siRNA treatment in T24 cells. Proteins associated with the AKT/GSK3β signalling pathway were also detected. GAPDH was used as an internal reference. D, Immunofluorescence staining of N‐cadherin and E‐cadherin, which are mesenchymal and epithelial markers, respectively, confirmed the alteration of EMT. *P < 0.05, **P < 0.01. The scale bar is 200 μm

Metastasis was the greatest challenge in clinical management of tumours, which was associated with cell motility. Our transwell invasion and migration assay revealed that knockdown of *PPARγ* could decrease the cell invasion and migration rate of BCa cells (Figure 3A; Figure S1E), which was confirmed by the statistical analysis (Figure 3B). In addition, proteins related to EMT were analysed by Western blot and IF staining, which showed that E‐cadherin, a marker of the epithelium, was up‐regulated, and the mesenchymal markers N‐cadherin and vimentin exhibited an especially strongly down‐regulation in the *PPARγ‐siRNA* group (Figure 3C,D; Figure S1H). Moreover, consistent with our KEGG pathway enrichment, the proteins associated with the AKT/GSK3β signalling pathway were altered, for example, p‐GSK3β was up‐regulated and p‐AKT was down‐regulated (Figure 3C). Furthermore, double IF staining showed that the fluorescence of p‐AKT (red) counterstained with PPARγ (green) was intensely decreased in the *PPARγ‐siRNA* group compared with NC group (Figure S1F).

### 3.4 Knockdown of PPARγ induced cell cycle arrest in G0/G1 phase but had no significant effect on apoptosis

**Figure 4:**
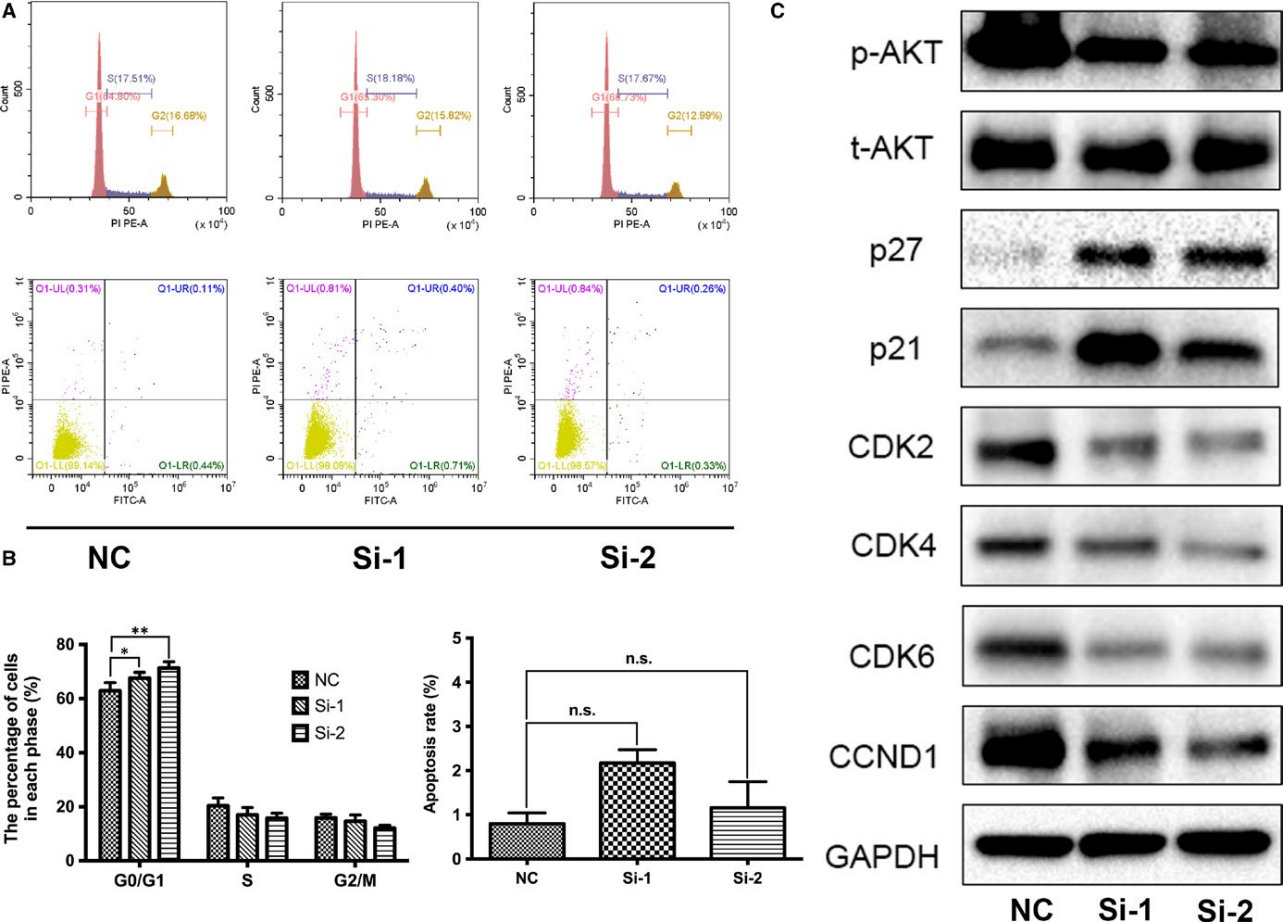
Down‐regulation of *PPARγ* induced cell cycle arrest in G0/G1 phase. A, Flow cytometry analysis of cell cycle and apoptosis in T24 cells with siRNA treatment. B, Statistical analysis of the cell cycle showed a significantly increased percentage of cell populations in G0/G1 phase. However, there was no obvious effect on apoptosis after the knockdown of *PPARγ*. C, Proteins related to G0/G1 phase in T24 cells were analysed by Western blotting. The upstream indicator p21/p27 was up‐regulated and phosphorylated/total AKT was down‐regulated. All values are presented as the mean ± SD from at least three independent research results. *P < 0.05, **P < 0.01, ns means no significance

Pathway enrichment analysis revealed that *PPARγ *was related to cell cycle, apoptosis and PI3K‐AKT signalling pathway (Figure 1E). Therefore, we used flow cytometry to assess the effect of PPARγ deficiency on the cell cycle in BCa cells (Figure 4A; Figure S1G), suggesting a significantly higher percentage of G0/G1 phase cell numbers (Figure 4B). Consistent with the cell cycle assay, the Western blot results indicated that G0/G1‐related proteins, such as CDK4, CDK6 and CCND1, were strongly reduced after knockdown of PPARγ (Figure 4C; Figure S1I). We also noted that the upstream indicators of the cell cycle pathway, such as p21 and p27, were up‐regulated, and phosphorylated/total AKT was down‐regulated (Figure 4C; Figure S1I). However, as shown by the flow cytometry analysis, PPARγ deficiency could not induce significant apoptosis in BCa cells (Figure 4A,B; Figure S1G).

### 3.5 The PPARγ antagonist GW9662 inhibited the motility and proliferation of BCa cells

**Figure 5:**
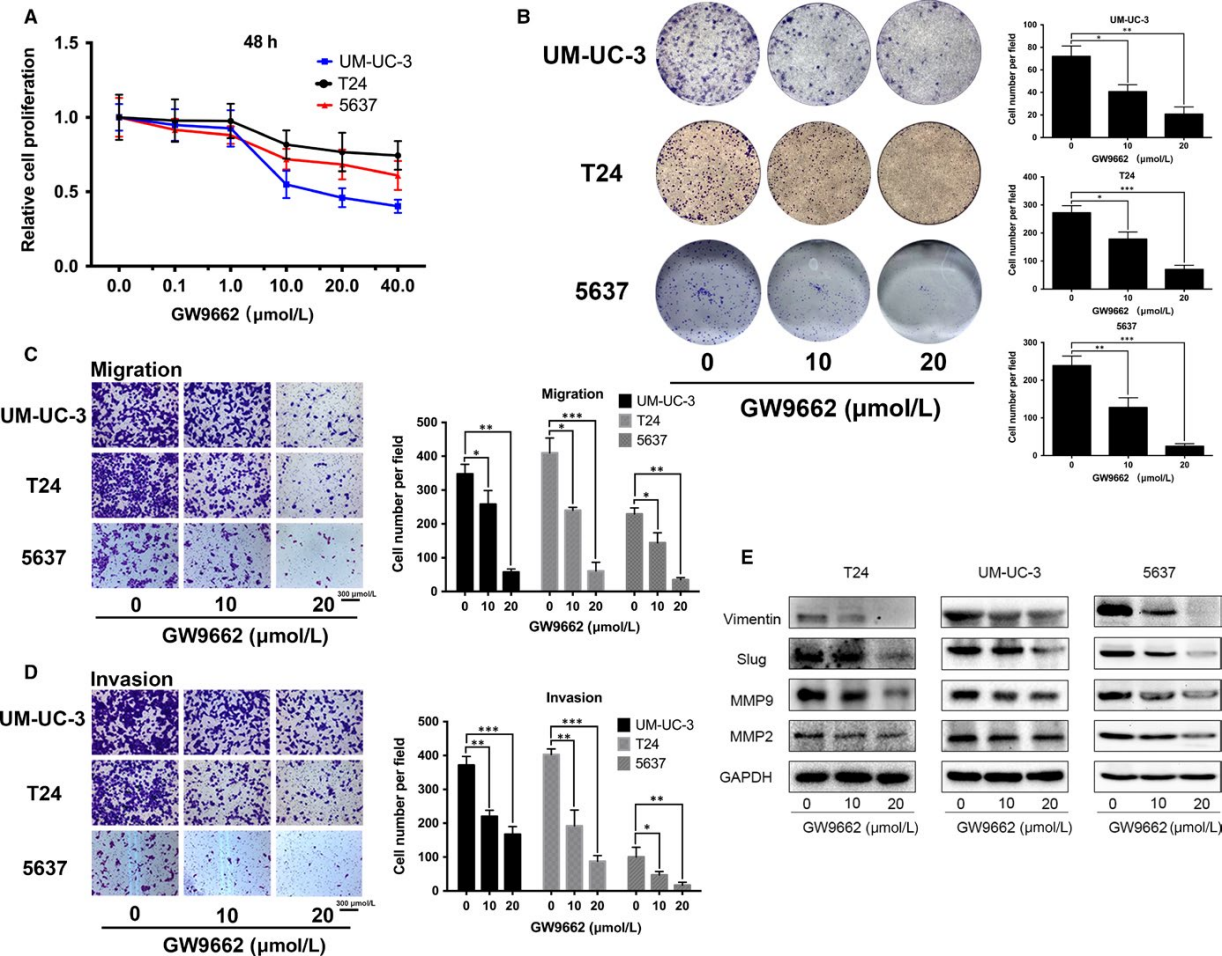
GW9662 inhibited the viability and motility of BCa cells. A, To determine the appropriate concentrations of GW9662, the proliferation of UM‐UC‐3 and T24 cells treated with GW9662 at different concentrations (0, 0.1, 1, 10, 20 and 40 μmol/L) were detected using the methyl thiazolyl tetrazolium assay. The results after treatment for 48 h are shown. B, Alterations of cell survival were detected using the clonogenic forming assay after treatment with GW9662. The colony number in each well was counted and statistically analysed. Cell types and drug concentrations are indicated. C, The migration assay showed a decreased number of migrated T24 and UM‐UC‐3 cells after treatment with GW9662, as confirmed by the statistical analysis. D, GW9662 treatment with different concentrations inhibited the invasion rate of BCa UM‐UC‐3 and T24 cells. The scale bar is indicated. E, Western blot analysis of vimentin, slug, MMP2 and MMP9 showed a considerable decrease after GW9662 treatment. GAPDH was used as internal reference. Data are presented as the mean ± SD. *P < 0.05, **P < 0.01, ***P < 0.001

To further study the effect of drug inhibitor of PPARγ protein on BCa cells, UM‐UC‐3, 5637 and T24 cells were treated with GW9662 at different concentrations (0, 0.1, 1, 10, 20 and 40 μmol/L) for different times and the MTT assay was conducted to evaluate the cell viability (Figure S2). The results showed a relatively reduced cell growth in a dose‐dependent manner. Considering the different cell inhibition and response to GW9662 (Figure 5A), in the following in vitro studies, cells were pre‐treated with GW9662 at 0 (control), 10 and 20 μmol/L for 48 hours in advance. Consistently, the clonogenic forming assay revealed a significant reduction in the colony counts in the GW9662‐treated group compared with the control group (Figure 5B). The transwell invasion and migration assay showed that GW9662 could decrease the cell migration (Figure 5C) and invasion rate of BCa cells (Figure 5D), and it was validated by the statistical analysis (Figure 5C,D). In accordance with the transwell assay, the Western blot assay showed an obvious down‐regulation of vimentin, Slug and MMP9 proteins involved in the EMT process (Figure 5E).

### 3.6 GW9662 suppressed BCa cell growth in vivo

**Figure 6:**
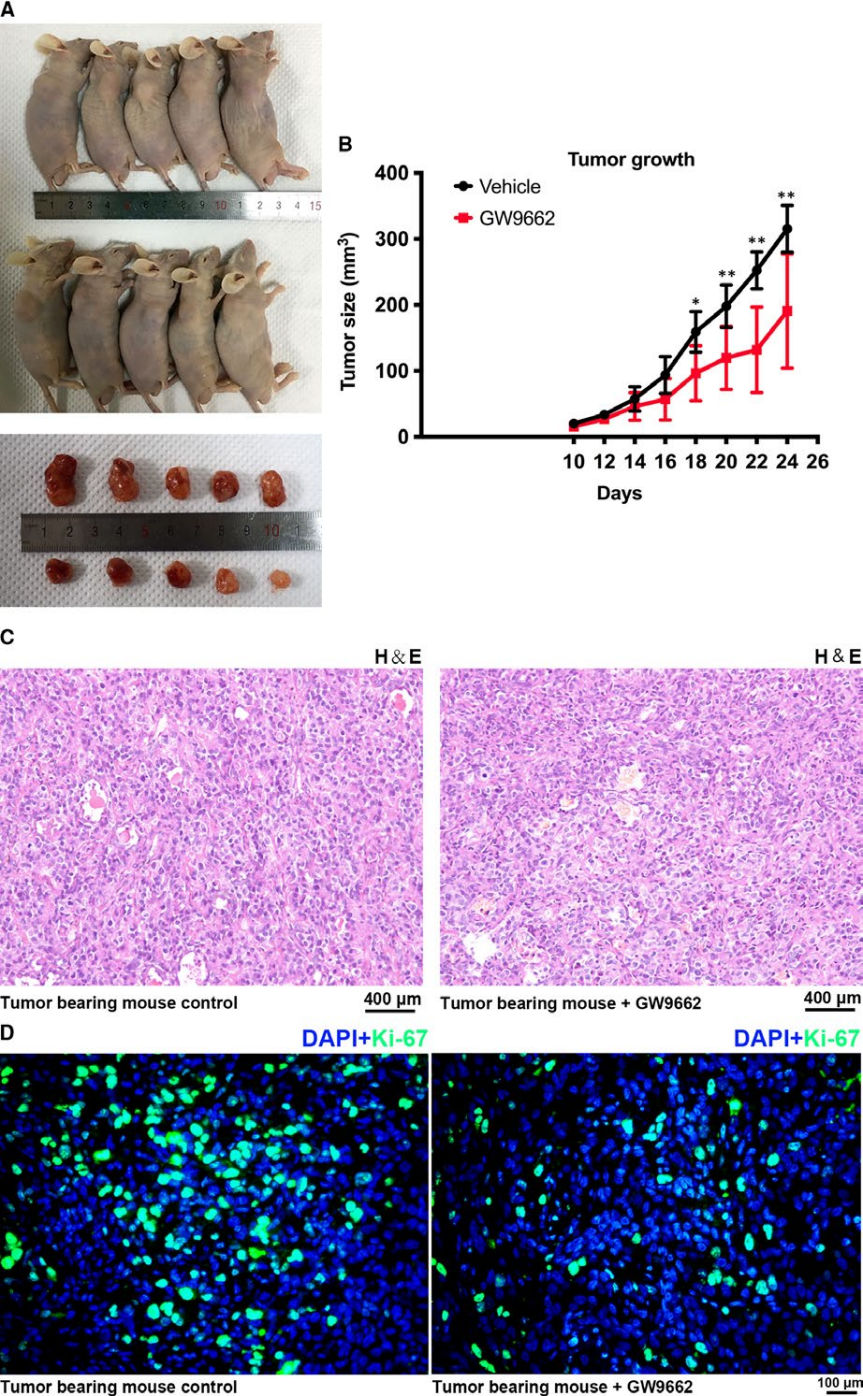
GW9662 inhibited BCa growth in vivo. A, BALB/c nude mice were subcutaneously injected with T24 cells and allowed to grow for 10 d. Then GW9662 (1 mg/kg) and vehicle were then intraperitoneally injected every other day for additional 14 d. After the mice were killed, tumour tissues were dissected. B, The tumour volume was calculated before each injection, and statistical analysis was carried out with the *t* test. The days after transplantation of T24 cells and the tumour size are indicated. *P < 0.05, **P < 0.01. C, Representative haematoxylin and eosin staining of tumour tissues dissected from the tumour‐bearing mice. The scale bar is indicated. D, The proliferation of T24 cells treated with GW9662 and vehicle in vivo was measured by immunofluorescence staining of Ki‐67 (green). Nuclei were stained with 4',6‐diamidino‐2‐phenylindole (DAPI; blue), and the scale bar is indicated

We established a mouse model by transplanting T24 cells (Figure 6A) to further evaluate the effect of PPARγ on BCa cell growth in vivo. The results suggested a significantly suppressed tumour growth in the GW9662 (1 mg/kg) injection group compared with the vehicle group (Figure 6B). The cancer tissues dissected from mice were stained with H&E (Figure 6C). Moreover, the IF results indicated that the positive staining for Ki‐67, which is an important marker of cell proliferation,^37^ was considerably lower in the GW9662‐injected group in comparison to the vehicle group (Figure 6D).

## 4 DISCUSSION

Our group has revealed a variety of potential biomarkers^32^, ^38^, ^39^ based on transcriptome data of normal bladder tissues vs BCa tissues.^34^, ^41^ Most of them are related to the PPAR signalling pathway, which could represent a connection between fatty acid/lipid metabolism and BCa.^33^ The PPARs, including the PPARα, PPARβ/δ and PPARγ subtypes encoded by different genes, are a family group of nuclear receptors.^42^ Recent studies have also shown significantly activation of the PPARγ signalling pathway in BCa, with a highly enriched *PPARγ* mRNA expression.^43^, ^44^ Consistently, in this study, we found an up‐regulated PPARγ expression in BCa both at mRNA and protein levels (Figure 1B,C). To investigate the effect of PPARγ on BCa, we generated a cell model with PPARγ deficiency using siRNA transfection. Indeed, we validated the knockdown efficiency of PPARγ at the gene expression and protein levels in the BCa T24 cell line (Figure 2A‐C, Figure S1A,B).

Moreover, our results revealed that the down‐regulation of PPARγ could inhibit the proliferation of T24 cells, which might be caused by cell cycle arrest in G0/G1 phase. Furthermore, proteins related to cell cycle regulation, CDK2/4/6 and CCND1, were all decreased after *si‐PPARγ* treatment. Cyclin‐dependent kinases (CDKs) are a family of protein kinases that play crucial roles in cell cycle regulation.^46^ Among them, CDK6 and CDK4 are responsible for releasing out from G0 phase to enter G1 phase,^47^, ^48^ while CDK2 protein plays a role in G1 phase.^49^ Down‐regulation of CDK2/4/6 can result in cell cycle arrest in G0/G1 phase,^50^ which could explain the results of the flow cytometry analysis (Figure 4A). P21 and p27, which have been reported as two important tumour suppressor proteins, play a significant role in inhibiting cell growth.^51^ After knockdown of PPARγ, we noted a significant up‐regulation of p21 and p27 and a down‐regulation of phosphorylated/total AKT. However, down‐regulation of PPARγ did not cause apoptosis in BCa cells, indicating that PPARγ deficiency may be not sufficient to induce cell death in BCa cells, partially because of the knockdown method instead of the knockout. Furthermore, we observed an inhibitory effect of PPARγ knockdown on invasion and migration, with up‐regulated E‐cadherin and down‐regulated N‐cadherin, vimentin, Slug, MMP2 and MMP9 proteins related to EMT, which has been demonstrated to play a crucial role in cancer cell invasion and migration.^52^ The association of vimentin and cytokeratin was also revealed in the transitional cell carcinoma genesis of urinary bladder patients, and thus could be a favourable marker in the early diagnosis of transitional BCa.^53^ Consistently, in this study, the potential BCa marker vimentin was reduced in BCa T24 cells treated with PPARγ deficiency. The phosphorylation of AKT at Ser9 could inhibit the activity of GSK‐3β.^54^ Consistently, in our study, we noted that phosphorylated/total AKT was down‐regulated and phosphorylated/total GSK3β was up‐regulated, which indicated that the alteration of EMT might be regulated via the AKT/GSK3β signalling pathway.

To better understand the effect of PPARγ on BCa, GW9662 was used for subsequent pharmacologic inhibition, a potent and pure antagonist of PPARγ.^31^ Recent studies have revealed that GW9662, with or without PPARγ ligands, could be a potential therapeutic strategy targeting glioblastoma stem cells.^55^, ^56^ In accordance with the knockdown assays, our results revealed that GW9662 could inhibit the proliferation of BCa 5637, T24 and UM‐UC‐3 cells in a time and dose‐dependent manner. The invasion and migration rate were also reduced in BCa cells treated with GW9662, with reversed EMT markers, which could suggest tumour malignancy.^57^, ^58^ However, we did not notice the same changes of AKT/GSK3β‐related proteins as in PPARγ knockdown assay. The reason might be that GW9662 treatment could cause multiple alterations of pathway‐related proteins, which could be caused by the drug effect that was different from PPARγ knockdown. And the deep mechanism still needs to be further studied. Interestingly, we found that GW9662 did not modulate the expression of PPARγ in BCa cells and the cell cycle was not arrested in G0/G1 phase as in the PPARγ knockdown assay, indicating that GW9662 and knockdown of PPARγ might regulate the cell cycle through a different pathway in BCa cells. Mehta et  al^59^ have also suggested that GW9662 could be mediated not by the direct association of PPARγ with GW9662 but through a different mechanism.

Furthermore, we established a T24‐transplanted BALB/c nude mouse model in which an intraperitoneal injection of GW9662 and vehicle as delivered. Significantly delayed tumour growth was observed in the GW9662 group compared with the vehicle group, as confirmed by IF staining of Ki‐67, a proliferation marker protein,^60^ indicating a growth inhibitory effect of GW9662 in vivo. Therefore, our results revealed that GW9662 could inhibit BCa cell proliferation in vivo and in vitro and down‐regulation of PPARγ could trigger cell cycle arrest, induce cell proliferation and decrease cell motility of BCa cells. Taken together, PPARγ is a promising therapeutic target in BCa and GW9662 has the potential to become a targeted therapeutic strategy against BCa.

## CONFLICT OF INTEREST

The authors declare no conflict of interests.

## AUTHORS' CONTRIBUTIONS

Conceptualization: KQ, GW, YX and XW; methodology, software and revision: SC and YW; validation: YX and XL; formal analysis, investigation and visualization: YX; resources: KQ and GW; data curation: GW; writing—original draft preparation: SC; writing—review and editing: KQ; project administration and funding acquisition: XW

## AVAILABILITY OF DATA AND MATERIALS

The datasets used and/or analysed during the current study are available from the corresponding author on reasonable request.
