# Supplementary figures and images for: PPARγ inhibition regulates the cell cycle, proliferation and motility of bladder cancer cells
Source: J Cell Mol Med. 2019 Mar 25;23(5):3724–36. doi: 10.1111/jcmm.14280 (PMC6484405; doi:10.1111/jcmm.14280)

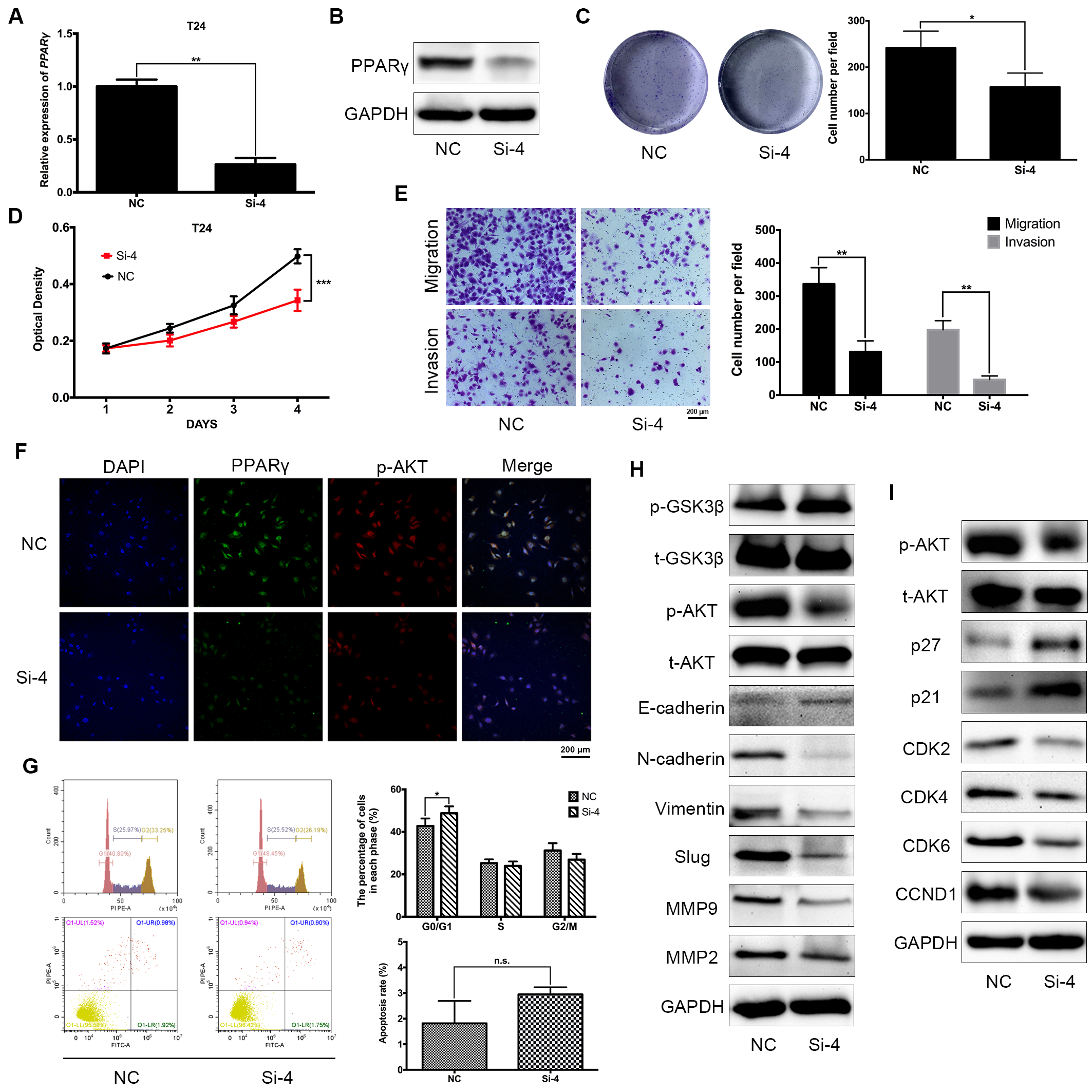

Supplement: Supplementary file 1 [file JCMM-23-3724-s001.tif]

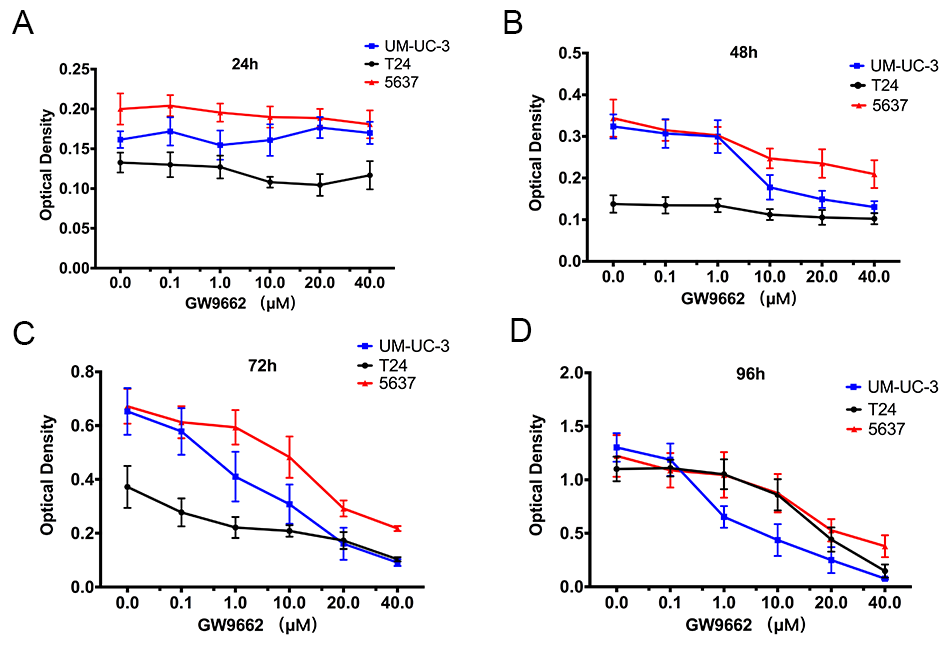

Supplement: Supplementary file 2 [file JCMM-23-3724-s002.tif]
