# Supplementary material for: PPARγ inhibition regulates the cell cycle, proliferation and motility of bladder cancer cells
Source: J Cell Mol Med. 2019 Mar 25;23(5):3724–36. doi: 10.1111/jcmm.14280 (PMC6484405; doi:10.1111/jcmm.14280)
Supplement: Supplementary file 3 [file JCMM-23-3724-s003.docx]

Table SI: Enrichment analysis of gene ontology

| Term | Overlap | Count | PValue | FDR |
| --- | --- | --- | --- | --- |
| REGULATION OF CELL PROLIFERATION | 171 | 1496 | 1.66E-69 | 7.70E-66 |
| POSITIVE REGULATION OF RESPONSE TO STIMULUS | 187 | 1929 | 4.40E-65 | 1.02E-61 |
| REGULATION OF CELL DEATH | 161 | 1472 | 3.71E-62 | 5.75E-59 |
| CELLULAR RESPONSE TO ORGANIC SUBSTANCE | 176 | 1848 | 1.82E-59 | 2.12E-56 |
| IMMUNE SYSTEM PROCESS | 180 | 1984 | 8.89E-58 | 8.27E-55 |
| POSITIVE REGULATION OF MULTICELLULAR ORGANISMAL PROCESS | 151 | 1395 | 3.04E-57 | 2.36E-54 |
| POSITIVE REGULATION OF BIOSYNTHETIC PROCESS | 167 | 1805 | 3.52E-54 | 2.05E-51 |
| RESPONSE TO OXYGEN CONTAINING COMPOUND | 146 | 1381 | 7.93E-54 | 4.10E-51 |
