# Supplementary material for: PPARγ inhibition regulates the cell cycle, proliferation and motility of bladder cancer cells
Source: J Cell Mol Med. 2019 Mar 25;23(5):3724–36. doi: 10.1111/jcmm.14280 (PMC6484405; doi:10.1111/jcmm.14280)
Supplement: Supplementary file 4 [file JCMM-23-3724-s004.docx]

**Figure S1.** Knockdown of PPARγ inhibited viability and motility of BCa T24 cells. PPARγ knockdown efficiency was validated by (A) qRT-PCR and (B) western blot analysis. (C) Clonogenic survival assay and statistical analysis was performed to detect the influence of PPARγ knockdown on cell survival. (D) MTT assay was used to test the cell viability. (E) Transwell invasion and migration assay for T24 cells with or without siRNA. (F) Double IF staining of p-AKT and PPARγ in siRNA-treated group and NC group. Nuclei were stained with DAPI (blue). (G) Flow cytometry analysis of cell cycle and apoptosis in T24 cells with or without siRNA treatment. Western blot analysis of (H) EMT-related and (I) cell cycle-related proteins. The scale bar and group is indicated. * p < 0.05, ** p< 0.01, *** p < 0.001, n.s. means no significance.

**Figure S2.** Evaluation of BCa cell growth and viability by GW9662 treatment. Cell proliferation was analyzed by MTT assay using 5637 (red, marked with solid triangle), T24 (black, marked with solid circle) and UM-UC-3 (blue, marked with solid square) cells treated with GW9662 at different concentration of 0, 0.1, 1, 10, 20 and 40 μM, cultured for 24 (A), 48 (B), 72 (C) and 96 h (D), to determine the appropriate concentrations of GW9662 treatment on BCa cells. The Y axis represents optical density.
