# Supplementary material for: PPARγ inhibition regulates the cell cycle, proliferation and motility of bladder cancer cells
Source: J Cell Mol Med. 2019 Mar 25;23(5):3724–36. doi: 10.1111/jcmm.14280 (PMC6484405; doi:10.1111/jcmm.14280)
Supplement: Supplementary file 5 [file JCMM-23-3724-s005.docx]

Table SII: List of KEGG pathway analysis

| Term | Overlap | Count | PValue | FDR |
| --- | --- | --- | --- | --- |
| PI3K_Akt_signaling_pathway_hsa04151 | 51 | 352 | 1.60E-23 | 4.15E-22 |
| MAPK_signaling_pathway_hsa04010 | 39 | 295 | 7.86E-17 | 6.81E-16 |
| Cytokine_cytokine_receptor_interaction_hsa04060 | 41 | 270 | 7.15E-20 | 1.24E-18 |
| Focal_adhesion_hsa04510 | 30 | 199 | 8.94E-15 | 6.64E-14 |
| Cellular_senescence_hsa04218 | 25 | 160 | 6.89E-13 | 4.48E-12 |
| Apoptosis_hsa04210 | 28 | 138 | 2.49E-17 | 2.59E-16 |
| FoxO_signaling_pathway_hsa04068 | 28 | 132 | 7.18E-18 | 9.33E-17 |
| Cell_cycle_hsa04110 | 22 | 124 | 1.20E-12 | 6.95E-12 |
